# Dietary inulin intake and age can significantly affect intestinal absorption of calcium and magnesium in rats: a stable isotope approach

**DOI:** 10.1186/1475-2891-4-29

**Published:** 2005-10-27

**Authors:** Charles Coudray, Mathieu Rambeau, Christine Feillet-Coudray, Jean Claude Tressol, Christian Demigne, Elyett Gueux, Andrzej Mazur, Yves Rayssiguier

**Affiliations:** 1 Centre de Recherche en Nutrition Humaine d'Auvergne, Unité Maladies Métaboliques et Micro-nutriments, INRA, Theix, 63122 St-Genès-Champanelle, France

**Keywords:** Inulin, intestinal absorption, status, calcium, magnesium, fermentation, stable isotope, age, rat

## Abstract

**Background:**

previous studies have shown that non-digestible inulin-type fructan intake can increase intestinal mineral absorption in both humans and animals. However, this stimulatory effect on intestinal absorption may depend on experimental conditions such as duration of fermentable fiber intake, mineral diet levels and animals' physiological status, in particular their age.

**Objectives:**

the aim of this study was to determine the effect of inulin intake on Ca and Mg absorption in rats at different age stages.

**Methods:**

eighty male Wistar rats of four different ages (2, 5, 10 and 20 months) were randomized into either a control group or a group receiving 3.75% inulin in their diet for 4 days and then 7.5% inulin for three weeks. The animals were fed fresh food and water *ad libitum *for the duration of the experiment. Intestinal absorption of Ca and Mg was determined by fecal monitoring using stable isotopic tracers. Ca and Mg status was also assessed.

**Results:**

absorption of Ca and Mg was significantly lower in the aged rats (10 and 20 mo) than in the young and adult rat groups. As expected, inulin intake increased Ca and Mg absorption in all four rat groups. However, inulin had a numerically greater effect on Ca absorption in aged rats than in younger rats whereas its effect on Mg absorption remained similar across all four rat age groups.

**Conclusion:**

the extent of the stimulatory effect of inulin on absorption of Ca may differ according to animal ages. Further studies are required to explore this effect over longer inulin intake periods, and to confirm these results in humans.

## Introduction

When non-digestible inulin-type fructans reach the large intestine, they are fermented by the local microflora and stimulate the growth of bifidobacteria and lactobacilli, which may have health-promoting functions [1-3]. Several studies have demonstrated that rats fed with prebiotic fructans absorbed more Ca and Mg than control rats, despite an increase in total fecal mass [4-6]. Indeed, the products of fructan fermentation can influence the intestinal absorption of Ca and Mg in many ways. Short-chain fatty acids (SCFA) are fermentation products that are responsible for lowering the pH of cecal content, which in turn increases mineral solubility, leading to improved mineral absorption [7]. SCFA can directly influence mineral absorption by forming complexes with the minerals, thereby increase their uptake by the intestinal cells [8,9]. It is thought that the bacterial metabolites (e.g. butyrate) can stimulate the intestinal epithelium and increase its absorptive capacity [10]. These various factors are closely linked to the nature of the prebiotic carbohydrates and to experimental conditions [7,11,12]. Inulin has been shown to have generally high and consistent effects on intestinal Mg absorption in both animals and humans [13], but the effects of inulin on calcium (Ca) absorption seem to be dependent on experimental conditions (dose of inulin, dietary Ca content, experiment duration, animal age and mineral requirements). In this study, we investigated the relationship between animal age and the stimulatory effect of inulin on intestinal absorption and retention of Ca and Mg using a stable isotope approach following short-term administration of inulin in rats aged from 2 to 20 months. This is the first time that the effect of inulin is studied in rats using a stable isotope approach

## Materials and methods

### Materials and reagents

The enriched Ca isotope (^44^Ca) as CaCO3 and the enriched Mg isotope (^25^Mg) as MgO were obtained from Chemgas, (Boulogne, France). The atomic abundances of these enriched isotopes were as follows: ^40^Ca = 3.41%, ^42^Ca = 0.09%, ^43^Ca = 0.03%, ^44^Ca = 96.45% ^46^Ca =< 0.01% ^48^Ca = 0.02% and ^24^Mg = 1.6%, ^25^Mg = 97.8%, ^26^Mg = 0.6%. HNO_3 _(ultrapure), Mg and beryllium standard solutions (1 g/L) were obtained from Merck (Darmstadt, Germany). All other chemicals were of the highest quality available. Distilled water was used throughout. A Perkin-Elmer 6100DRC system (Perkin-Elmer Instruments, Courteboeuf, France) equipped with a Meinhard nebulizer was used for isotopic measurement, and a Perkin Elmer AA800 (Perkin Elmer Instruments, Courteboeuf, France) was used for total Mg measurement.

### Animals and diets

Eighty male Wistar rats aged 2, 5, 10 or 20 months were purchased from Janvier (Le Genest Saint Ile, France). They were fed a commercial pellet diet (Ssniff R/S-breeding – until 3 mo, then Ssniff R/S maintenance from 3 to 24 mo age). Two groups were formed for each age bracket to receive either a control diet or a semi-purified diet containing inulin until the end of the experiment. The composition of these two diets is given in Table 1. Tested inulin was purchased from Orafti, Tienen, Belgium (Raftaline^®^). The target Ca and Mg levels in these diets were 5000 mg Ca/kg and 500 mg Mg/Kg diet. Powder diet (100 g) was made up with 100 ml of distilled water to form a kind of semi-liquid food prepared on-site each day. Chemical analysis of the diets offered confirmed the expected Ca and Mg contents in the experimental diets: 5107 mg Ca/kg and 5050 mg Ca/kg, and 495 mg Mg/kg and 514 mg Mg/kg in the control and inulin diets, respectively. Chemical analysis showed that the inulin contained approximately 40 mg Ca/kg and less than 1 mg Mg/kg. Dietary inulin level was maintained at 3.75% during the first 4 days and then 7.5% from day 5 until the end of the experiment. The 8 rat groups were given fresh food and water daily, made available *ad libitum*. Food consumption and body weight were recorded weekly. Throughout the experiment, the rats were housed two per cage (wire-bottomed to limit coprophagy) in a temperature-controlled room (22°C) with dark period from 08:00 pm to 08:00 am. Total experiment duration was 30 days. All procedures complied with the Institute's ethical guidelines on the care and use of laboratory animals.

**Table 1:** Diet composition (g/kg) during the experiment

	**Control diet**	**Inulin diets**	
		3.75%	7.5%
**Wheat starch**	**650**	**612.5**	**575**
Casein	200	200	200
Corn oil	50	50	50
Cellulose	50	50	50
Mineral mix (AIN 1993)a	35	35	35
Vitamin mix (AIN 1993)b	10	10	10
DL-Methionine	3	3	3
Choline bi-tartrate	2	2	2
**Inulin**	**0**	**37.5**	**75**

a: Mineral mix AIN 1993 ensures the following mineral levels in the diets (mg/kg): Na, 1020; K, 3600; P, 4000; Ca, 5000; Mg, 500; Zn, 30; Fe, 35; Cu, 6; Mn, 54; Se, 0.1; I, 0.2; Cr, 2.

b: Vitamin mix AIN 1993 ensures the following mineral levels in the diets (mg/kg): thiamine, 6; riboflavine, 6; pyridoxine, 7; nicotinic acid, 30; calcium pantothenate, 16; folic acid, 2; D-biotin: 0.2; and (μg/kg) cyanocobalamine (vitamin B12), 10; vitamin K, 50; and (IU/kg) vitamin A, 4000; vitamin E, 50; vitamin D, 1000.

### Preparation of stable isotope solution

215 mg of the ^44^Ca (in carbonate form = 508 mg) and 255 mg of the ^25^Mg (in oxide form = 412 mg) were first individually moistened with 2 ml of distilled water. One ml of HCl 12 N (ultrapure) was added to the ^44^Ca suspension and two ml of HCl 12 N was added to the ^25^Mg suspension to transform the carbonate and the oxide into soluble chlorides of Ca and Mg, respectively. Each solution was then diluted with 50 ml of distilled water, both solutions were then mixed, and pH was adjusted to between 3 and 6 with 1 N sodium hydroxide solution. The resulting study solution was then completed to 150 ml with distilled water and maintained for several days at +4°C until utilization. Total and isotopic Ca and Mg contents were checked before use.

The rats were transferred to metabolic cages and housed individually three days before the beginning of the isotopic balance study to allow them to adapt to their new environment. Animals received by gavage about 1.7 ml of isotopic solution. The urine and faeces of each rat were quantitatively collected for four consecutive days, and excreted isotopes in these two media and in the gavage solution were quantitatively determined by ICP/MS, as described below.

### Sampling procedures

The rats were sacrificed just after the dark period (between 08:00 am and 10:00 am), i.e. at a time when cecal fermentation was still very active. After anesthesia (40 mg sodium pentobarbital/kg body weight), blood was withdrawn from the abdominal aorta, placed into tubes containing sodium heparin and centrifuged at 1,000 *g *for 10 minutes. Plasma samples were stored at 4°C for mineral analysis. The cecum, complete with contents, was removed and weighed (total cecal weight). The cecal wall was flushed clean with ice-cold saline, blotted on filter paper, and weighed (cecal wall weight). For each rat, duplicate samples of cecal contents were collected into 2 ml microfuge tubes and immediately frozen at -20°C until analysis. The pH of cecal content was determined on site using a Sentron pH-system 1001 portable pH-meter (Sentron Europe B.V. Ac Roden, The Netherlands). Supernatants of the digestive contents were obtained by centrifuging one of the two microfuge tubes at 20,000 *g *for 10 minutes at 4°C, and then frozen until analysis. One tibia was also sampled for Ca and Mg analysis.

### Analytical procedures

Ca and Mg concentrations were determined in the plasma and urine after adequate dilution into 0.1% (w/v) lanthanum chloride. Diet aliquots, fecal materials and tibia were dry-ashed (10 hours at 500°C) and dissolved with concentrated HNO_3 _and H_2_O_2 _on a heating plate until complete decoloration. The resulting mineral solutions were set at 10 ml with water and adequately diluted in 0.1% lanthanum chloride. Mineral concentrations were measured by atomic absorption spectrophotometry (on a Perkin-Elmer AA800) at wavelengths of 422 nm for Ca and 285 nm for Mg.

For isotopic ^44^Ca and ^25^Mg determination, samples were appropriately diluted before analysis using 1% HNO_3_. Ca and Mg concentration and isotope ratios were determined by ICP-MS using Ca and Mg as external standard and beryllium as internal standard. The instrument operating conditions were set as follows after optimization with a solution of 1 μg indium/l: RF Power = 1050 W, Nebulizer Ar flow rate = 0.79 L/min, Auxiliary Ar flow rate = 1.2 L/min, Outer Ar flow rate = 15 L/min. Data acquisition parameters were set as follows: Sweeps/reading = 50, Readings/replicate = 1, Number of replicates = 3, Dwell time = 50 ms for ^24^Mg, 75 ms for ^9^Be, ^25^Mg, ^26^Mg, and ^44^Ca, 150 ms for ^42^Ca and 300 ms for ^43^Ca, Scanning mode = peak hopping. DRC operating conditions (for ^42^Ca, ^43^Ca and ^44^Ca) were as follows: Cell Gas A Flow Rate = 0.5 L ammonia/min, RPa = 0, and RPq = 0.45.

Cecal SCFA concentrations, including acetic, propionic and butyric acid, were determined by gas-liquid chromatography on portions of supernatant fractions of cecal contents as previously described [14].

### Calculations

Ca and Mg each have different stable isotopes with the following natural abundances: ^40^Ca = 96.941%, ^42^Ca = 0.647%, ^43^Ca = 0.135%, ^44^Ca = 2.086% ^46^Ca = 0.004% ^48^Ca = 0.187% and ^24^Mg = 78.99%, ^25^Mg = 10.00% and ^26^Mg = 11.01% [15]. ^44^Ca and ^25^Mg isotopic enrichments were obtained, respectively, from the following equations: (^44^Ca/^43^Ca measured ratio - ^44^Ca/^43^Ca baseline ratio)/(^44^Ca/^43^Ca baseline ratio) and (^25^Mg/^26^Mg measured ratio - ^25^Mg/^26^Mg baseline ratio)/(^25^Mg/^26^Mg baseline ratio).

Non-absorbed ^44^Ca and ^25^Mg isotopes in the fecal or urine samples (coming only from the ^44^Ca or ^25^Mg isotope labels) were calculated as follows:

for ^44^Ca (mg) = (total fecal or urine Ca (mg) × (natural abundance ^44^Ca × enriched ^44^Ca)/(1 + (natural abundance ^44^Ca × enriched ^44^Ca);

for ^25^Mg (mg) = (total fecal or urine Mg (mg) × (natural abundance ^25^Mg × enriched ^25^Mg)/(1 + (natural abundance ^25^Mg × enriched ^25^Mg)).

Calculations were also made directly from ICP-MS data. The two modes of calculation give the same results when the ICP-MS quantitative procedure is used [16].

Intestinal absorption of ^44^Ca and ^25^Mg was then calculated as administered ^44^Ca or ^25^Mg - ^44^Ca or ^25^Mg excreted in the feces, and retention of ^44^Ca and ^25^Mg was calculated as administered ^44^Ca or ^25^Mg - ^44^Ca or ^25^Mg excreted in the feces and in the urine.

Total cecal SCFA content (μmol/cecum) was calculated as the supernatant SCFA concentration (μmol/ml) × cecal water (ml/cecum).

Soluble Ca and Mg levels in the cecal contents were determined on the supernatant concentration (μg/ml), and soluble Ca and Mg contents per cecum were calculated as (μg Ca/ml or μg Mg/ml) × cecal water (ml).

### Data analysis

Values are given as means ± SD, and data were tested by 2-way ANOVA using the General Linear Models procedure of the Super ANOVA package (Abacus, Berkeley, CA). Post-hoc comparisons were performed using Fisher's least significant difference procedures. Differences of *p *< 0.05 were considered statistically significant. Simple linear correlation analysis was used to assess the relationships between intestinal absorption of Ca and Mg and other relevant parameters. Values of *p *< 0.05 were considered statistically significant.

## Results

### Food intake and growth rate

Inulin intake at the dose of 7.5% showed only a tendency to decrease animal food intake in this study. The slight decrease in food intake in inulin-fed rats led to a non-significantly lower growth rate (*p *< 0.10) towards the end of the experiment in inulin-fed rats compared to controls. The lower calorific value of the inulin diets (-4%) compared to the control diets may also be responsible for this reduced weight gain. In addition, food intake decreased significantly with increasing age, as expected (data not shown).

### Cecal fermentation parameters and total and cecal soluble Ca and Mg levels (table 2)

**Table 2:** Effect of age and inulin intake and their interaction on cecum fermentation parameters and cecal Ca and Mg levels in rats

	**Cont 3 Mo**	**Cont 6 Mo**	**Cont 11 Mo**	**Cont 21 Mo**	**Inulin 3 Mo**	**Inulin 6 Mo**	**Inulin 11 Mo**	**Inulin 21 Mo**	**inulin**	**age**	**interaction**
**Cecal content pH**	6.92 ± 0.24	6.87 ± 0.17	6.72 ± 0.58	6.62 ± 0.31	5.71 ± 0.58	5.41 ± 0.22	5.64 ± 0.37	5.57 ± 0.22	<0.0001	NS	NS
**Cecal content, g**	2.20 ± 0.35	2.34 ± 0.68	2.53 ± 0.97	2.86 ± 0.74	6.18 ± 1.68	6.46 ± 1.57	7.09 ± 2.31	7.10 ± 1.91	<0.0001	NS	NS
**Cecal wall, g**	0.87 ± 0.07	1.11 ± 0.22	1.32 ± 0.29	1.25 ± 0.15	1.80 ± 0.37	2.32 ± 0.29	2.51 ± 0.39	2.46 ± 0.30	<0.0001	NS	NS
**Acetate, μmol/cecum**	22.4 ± 6.3	24.5 ± 9.4	24.4 ± 11.8	28.0 ± 8.5	49.2 ± 22.3	70.4 ± 16.7	54.3 ± 17.3	63.5 ± 11.4	<0.0001	0.033	NS
**Propionate, μmol/cecum**	5.49 ± 1.48	6.31 ± 2.20	5.11 ± 2.10	6.11 ± 1.72	15.56 ± 12.29	12.84 ± 5.04	9.96 ± 4.86	11.26 ± 3.92	<0.0001	NS	NS
**Butyrate, μmol/cecum**	9.90 ± 2.70	8.63 ± 3.10	6.37 ± 2.78	8.01 ± 3.38	48.13 ± 25.12	69.51 ± 32.50	39.98 ± 18.42	43.15 ± 8.63	<0.0001	0.018	0.037
**Total SCFA, μmol/cecum**	37.8 ± 9.1	39.5 ± 13.5	35.9 ± 15.9	42.2 ± 12.6	112.9 ± 50.8	152.8 ± 40.0	104.2 ± 27.7	117.9 ± 17.3	<0.0001	0.024	0.049

Values are mean ± SD, n = 10 animals.

The rats were received inulin (7.5%) for 4 weeks before cecal parameters assessment.

As expected, inulin intake significantly increased cecal wall weight and cecal content and significantly decreased the pH of cecal content. These variables did not change with rat age. In addition, inulin intake considerably increased the individual and total pools of SCFA in the cecal contents (*p *< 0.0001). The effect of age on these SCFA pools was less clear. No significant age-related difference was observed amongst the control group rats, whereas in the inulin-fed group, the intestinal bacteria produced higher acetate, butyrate and total SCFA in the rats aged 10 mo than in the three other groups (*p *< 0.05).

### Intestinal absorption and retention of calcium (table 3)

**Table 3:** Effect of age and inulin intake and their interaction on intestinal absorption and retention of Ca in rats

	**Cont 3 Mo**	**Cont 6 Mo**	**Cont 11 Mo**	**Cont 21 Mo**	**Inulin 3 Mo**	**Inulin 6 Mo**	**Inulin 11 Mo**	**Inulin 21 Mo**	**inulin**	**age**	**interaction**
**Administered ^44^Ca, μg**	1637 ± 46	1610 ± 14	1602 ± 17	1605 ± 24	1593 ± 19	1614 ± 19	1621 ± 25	1626 ± 22	NS	NS	<0.0005
**Fecal ^44^Ca enrichment, %**	12.5 ± 3.3	17.3 ± 2.4	20.2 ± 4.3	18.7 ± 2.8	10.9 ± 4.2	17.8 ± 6.2	17.4 ± 2.1	21.2 ± 3.1	NS	<0.0001	NS
**Fecal ^44^Ca level, μg/g**	112 ± 30	182 ± 35	218 ± 45	204 ± 38	76 ± 30	152 ± 49	163 ± 26	188 ± 26	<0.0001	<0.0001	NS
**Fecal ^44^Ca excretion, μg**	856 ± 224	1139 ± 153	1389 ± 96	1366 ± 115	541 ± 223	926 ± 142	1207 ± 195	1192 ± 142	<0.0001	<0.0001	NS
**Intestinal ^44^Ca absorption, μg**	781 ± 206	471 ± 153	213 ± 90	239 ± 117	1052 ± 222	689 ± 142	413 ± 202	434 ± 143	<0.0001	<0.0001	NS
**Intestinal ^44^Ca absorption, %**	47.8 ± 12.9	29.3 ± 9.4	13.3 ± 5.6	14.9 ± 7.3	66.1 ± 13.9	42.7 ± 8.8	25.4 ± 12.4	26.7 ± 8.7	<0.0001	<0.0001	NS
**Urinary ^44^Ca enrichment, %**	17.4 ± 6.5	20.6 ± 6.7	14.8 ± 3.1	16.9 ± 3.9	13.7 ± 3.3	17.5 ± 6.7	18.6 ± 4.2	18.5 ± 4.1	NS	NS	0.0569
**Urinary ^44^Ca excretion, μg**	15.3 ± 5.9	14.8 ± 6.0	25.8 ± 10.7	28.1 ± 7.9	23.5 ± 7.2	21.8 ± 9.9	49.0 ± 11.8	40.3 ± 12.5	<0.0001	<0.0001	0.036
**^44^Ca retention, μg**	765 ± 204	456 ± 153	188 ± 92	212 ± 113	1029 ± 219	667 ± 135	364 ± 203	394 ± 145	<0.0001	<0.0001	NS
**^44^Ca retention, %**	46.9 ± 12.8	28.3 ± 9.5	11.7 ± 5.7	13.2 ± 7.1	64.6 ± 13.8	41.3 ± 8.3	22.4 ± 12.4	24.2 ± 8.9	<0.0001	<0.0001	NS

Values are mean ± SD, n = 10 animals.

The rats were given ^44^Ca after 14 days of inulin intake (7.5%), and fecal non-absorbed ^44^Ca isotope was determined in a 4d feces and urine pools.

The amount of gavaged ^44^Ca was about 1.60 mg/rat, which led to a fecal ^44^Ca enrichment of 10% to 20% in the 4-day feces pool. Fecal ^44^Ca excretion expressed as mg/g of feces or as mg/day increased significantly with age. Consequently, net (mg) and relative (%) ^44^Ca absorption were significantly lower in the aged rats than in the young adult or adult rats. In addition, urinary ^44^Ca excretion (mg) increased significantly with age. Consequently, net (mg) and relative (%) ^44^Ca retention were considerably lower in the aged rats than in the young adult or adult rats. Inulin intake significantly decreased fecal ^44^Ca excretion, expressed as μg/g of feces or as μg, in all groups. Consequently, inulin intake significantly increased net (mg) and relative (%) ^44^Ca absorption. Moreover, inulin intake increased urinary ^44^Ca excretion (mg). Lastly, inulin intake significantly increased net (mg) and relative (%) ^44^Ca retention in the four age-related groups compared to the control diet groups.

### Intestinal absorption and retention of magnesium (table 4)

**Table 4:** Effect of age and inulin intake and their interaction on intestinal absorption and retention of Mg in rats

	**Cont 3 Mo**	**Cont 6 Mo**	**Cont 11 Mo**	**Cont 21 Mo**	**Inulin 3 Mo**	**Inulin 6 Mo**	**Inulin 11 Mo**	**Inulin 21 Mo**	**inulin**	**age**	**interaction**
**Administered ^25^Mg, μg**	2553 ± 71	2511 ± 22	2499 ± 26	2504 ± 38	2485 ± 30	2518 ± 30	2527 ± 39	2536 ± 35	NS	NS	<0.0005
**Fecal ^25^Mg enrichment, %**	47.9 ± 7.1	51.9 ± 7.4	54.6 ± 12.3	54.1 ± 8.1	33.9 ± 20.3	41.0 ± 19.9	47.4 ± 13.9	65.7 ± 17.6	NS	0.0007	0.027
**Fecal ^25^Mg level, μg/g**	149 ± 26	184 ± 34	205 ± 44	204 ± 38	51 ± 33	70 ± 35	91 ± 31	120 ± 35	<0.0001	<0.0001	NS
**Fecal ^25^Mg excretion, μg**	1138 ± 201	1157 ± 229	1311 ± 200	1366 ± 177	358 ± 224	430 ± 206	673 ± 205	757 ± 184	<0.0001	<0.0001	NS
**Intestinal ^25^Mg absorption, μg**	1415 ± 187	1354 ± 225	1188 ± 199	1137 ± 175	2127 ± 224	2087 ± 208	1855 ± 232	1780 ± 186	<0.0001	<0.0001	NS
**Intestinal ^25^Mg absorption %**	55.5 ± 7.5	54.0 ± 9.0	47.5 ± 8.0	45.4 ± 7.0	85.6 ± 8.9	82.9 ± 8.2	73.3 ± 8.4	70.2 ± 7.2	<0.0001	<0.0001	NS
**Urinary ^25^Mg enrichment, %**	29.3 ± 2.7	29.1 ± 3.5	28.0 ± 3.7	28.2 ± 2.32	34.2 ± 3.8	35.5 ± 2.4	33.5 ± 3.7	36.7 ± 6.5	<0.0001	NS	NS
**Urinary ^25^Mg excretion, μg**	398 ± 64	323 ± 36	298 ± 80	292 ± 88	699 ± 91	792 ± 167	633 ± 146	551 ± 164	<0.0001	0.003	NS
**^25^Mg retention, μg**	1017 ± 189	1031 ± 225	890 ± 179	845 ± 142	1428 ± 216	1295 ± 192	1221 ± 242	1228 ± 165	<0.0001	0.011	NS
**^25^Mg retention, %**	39.8 ± 7.3	41.1 ± 9.0	35.6 ± 7.1	33.7 ± 5.7	57.8 ± 8.9	51.5 ± 7.8	48.3 ± 9.2	48.4 ± 6.5	<0.0001	0.008	NS

Values are mean ± SD, n = 10 animals.

The rats were given ^25^Mg after 14 days of inulin intake (7.5%), and fecal non-absorbed ^25^Mg isotope was determined in a 4d feces and urine pools.

The amount of gavaged ^25^Mg was about 2.50 mg/rat, which led to a fecal ^25^Mg enrichment of 35% to 65% in the 4-day feces pool. Fecal ^25^Mg excretion expressed as mg/g of feces or as mg increased significantly with age. Consequently, net (mg) and relative (%) ^25^Mg absorption were significantly lower in the aged rats than in the young adult or adult rats. In addition, urinary ^25^Mg excretion (mg) increased significantly with age. Consequently, net (mg) and relative (%) ^25^Mg retention were significantly lower in the aged rats than in the young adult or adult rats. As expected, inulin intake significantly decreased fecal ^25^Mg excretion, expressed as μg/g of feces or as μg, in all groups. Consequently, inulin intake significantly increased net (mg) and relative (%) ^25^Mg absorption. Similarly, inulin intake increased urinary ^25^Mg excretion (mg). However, inulin intake led to significantly higher net (mg) and relative (%) ^25^Mg retention in all four groups compared to the control diet.

### Calcium and magnesium status (table 5)

**Table 5:** Effect of age and inulin intake and their interaction on status biomarkers of Ca and Mg in rats

	**Cont 3 M**	**Cont 6 M**	**Cont 11 M**	**Cont 21 M**	**Inulin 3 M**	**Inulin 6 M**	**Inulin 11 M**	**Inulin 21 M**	**inulin**	**age**	**interaction**
**Plasma Ca, mg/L**	98 ± 4	98 ± 5	95 ± 6	100 ± 5	102 ± 5	99 ± 4	98 ± 3	100 ± 4	0.0601	0.0619	NS
**Tibia weight, mg dw**	480 ± 42	630 ± 80	717 ± 89	630 ± 93	489 ± 66	617 ± 66	841 ± 92	648 ± 47	0.042	<0.0001	0.023
**Bone Ca, mg/g dw**	207 ± 21	214 ± 15	216 ± 15	215 ± 21	205 ± 13	215 ± 18	202 ± 14	228 ± 7	NS	0.018	0.0639
											
**Plasma Mg, mg/L**	17.9 ± 1.1	17.7 ± 1.1	17.2 ± 1.0	16.9 ± 1.3	17.6 ± 1.1	17.9 ± 1.3	18.2 ± 1.5	18.2 ± 1.7	0.0570	NS	NS
**Erythrocyte Mg, mg/L**	45.4 ± 3.8	44.2 ± 4.7	42.5 ± 3.4	43.4 ± 3.0	44.9 ± 4.9	43.8 ± 2.5	44.3 ± 3.6	43.8 ± 3.9	NS	NS	NS
**Bone Mg, mg/g dw**	3.92 ± 0.10	3.79 ± 0.08	3.72 ± 0.08	3.76 ± 0.08	3.91 ± 0.10	3.72 ± 0.07	3.73 ± 0.09	3.72 ± 0.09	NS	<0.0001	NS

Values are mean ± SD, n = 10 animals.

Mean plasma Ca varied from 95 to 102 mg/L, showing a tendency to increase with inulin intake (+2%, *p *= 0.0601) and to decrease with increasing age (-1%, *p *= 0.0619). Mean bone Ca varied from 202 to 228 mg/g dry weight, and was unaffected by inulin intake. However, mean bone Ca increased significantly with increasing age. Mean plasma Mg varied from 16.9 to 18.2 mg/L, showing a tendency to increase with inulin intake (+3%, *p *= 0.0570). However, mean plasma Mg was not modified by age. Plasma Mg increased in the inulin-fed aged rats (+6.7%), whereas there was no increase in the young and adult rats (-0.3%). Mean red blood cell Mg levels varied from 42.5 to 45.4 mg/L and remained unchanged when age increases or under inulin intake. Mean bone Mg levels varied from 3.72 to 3.92 mg/g dry weight, decreasing significantly with aging (*p *< 0.0001). However, mean bone Mg was unaffected by inulin intake.

## Discussion

Previous studies have repeatedly shown that intake of different inulin-type fructans can variably increase mineral intestinal absorption in humans and animals [4,5,17-19]. Indeed, inulin-type fructans strongly and consistently increase intestinal Mg absorption [12], whereas their effect on Ca absorption seems to be dependent on experimental conditions such as inulin type, dietary Ca levels, duration of fructan intake [20-22] and the animals' physiological state, particularly age. It is well known that the absorption mechanisms of Ca and Mg differ considerably [23,24], which may explain the observed differences between these two minerals in terms of inulin effect. In this study, we investigated the enhancing effect of fructan intake on Ca and Mg intestinal absorption and balance in rats of different ages.

### 1 – Effect of animal age and inulin intake on Ca absorption

Our results clearly showed that aged rats exhibited less efficient intestinal absorption and retention of Ca. ^44^Ca absorption ranged from 48% without inulin to 66% under inulin intake in the young and adult rats and from 15% without inulin to 27% under inulin intake in the old and very old rats. This decline in Ca absorption with age is not new, and has already been reported in animal and human studies [25-27] and is largely confirmed in this study. This decline is primarily due to an energy- and vitamin D-dependent Ca transport component in the elderly [28]. Our results clearly showed that inulin intake increased the efficiency of Ca intestinal absorption and retention. The mean ^44^Ca absorption in the four rat control groups was 26.3% compared to 40.2% in the four inulin-fed groups, with an overall increase in ^44^Ca absorption of 53%. These results are in agreement with literature data showing that the effect of inulin on Ca absorption seems to be optimal in the early weeks, then decreasing gradually with experiment duration [20,29,30]. One possible explanation for this phenomenon is a down-regulation of the active pathway of intestinal Ca absorption after several weeks of feeding inulin, as previously reported [31,32].

### 2 – Effect of animal age and inulin intake on Mg absorption

Our results showed that aged rats exhibited less efficient intestinal absorption and retention of Mg. ^25^Mg absorption ranged from 56% without inulin to 86% under inulin intake in the young and adult rats and from 45% without inulin to 70% under inulin intake in the old and very old rats. This decline in Mg absorption with age is not well documented in the literature in either animal or human studies. Few, if any, incomplete studies have reported an age effect on Mg absorption [33-35], and the results are inconsistent. Hence, to our knowledge, this is the first robust report to clearly show that Mg absorption decreases with age in the rat. Although Mg absorption is generally described as a passive phenomenon, one component of this absorption remains under hormonal control [36,37], which may explain the observed results. Our results clearly showed that inulin intake considerably increased Mg intestinal absorption and retention efficiency. Mean ^25^Mg absorption in the four rat control groups was 50.6%, compared to 78.0% in the four corresponding inulin-fed rat groups, with an overall increase in ^25^Mg absorption of 54%. These results are in agreement with literature data showing that inulin intake considerably increases Mg absorption in animals and humans (see recent review [13]).

### 3 – Modulation of the stimulatory inulin effect on Ca and Mg absorption with rat age

Since Ca absorption is generally well controlled, the observed absorption increase under inulin intake may be down-regulated (known as a feed-back phenomenon) in adult rats. Thus, given that Ca absorption is low and the adaptative phenomenon less well controlled in aged rats, we hypothesized that inulin intake would lead to a much greater increase in Ca absorption in aged rats than in the young or adult rats. Conversely, since Mg absorption is only weakly controlled with a generally consistent increase under inulin intake in adult rats, we hypothesized that inulin intake would increase Mg absorption in both aged rats and young or adult rats to the same extent.

**Figure 1:**
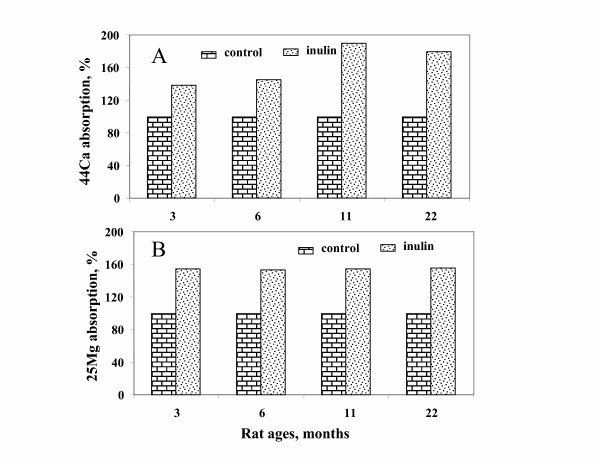
**Stimulatory effect of dietary inulin intake on intestinal absorption of ^44^Ca and ^25^Mg in rats of different ages**. Intestinal ^44^Ca absorption (A) and ntestinal ^25^Mg absorption (B) in the control groups was normalized to 100% for each age group. The stimulatory effect of inulin (%) for a given age group was calculated as follows: 100* (intestinal absorption in the inulin-fed age group/intestinal absorption in the same age group without inulin). The rats were given ^44^Ca and ^25^Mg after 14 days of inulin intake (7.5%), and fecal non-absorbed isotopes were determined in a 4d feces pool.

The relative increase in ^44^Ca absorption under inulin intake was 41.5% and 84.5% in the younger (3 and 6 mo old) and older rats (11 and 21 mo old), respectively (figure 1). Although these increase percents are numerically more important in the older rats than in the younger rats, there was no statistically significant interaction between age and inuline. It is highly possible that the number of animals used in this experiment was not enough to reach significant level. Furthermore, the relative increase in ^25^Mg absorption under inulin intake was 53.5% and 54.5% in the younger and older rats, respectively (figure 1). This indicates that the stimulatory effect of inulin on ^25^Mg absorption was not age-dependent. It is possible that inulin intake may lead to a higher increase in ^44^Ca absorption in the older rats than in the younger rats, whereas inulin intake leads to a similar increase in ^25^Mg absorption in young, adult and aged rats, thus confirming the hypothesis we formulated for this study.

In conclusion, as expected, our results confirmed that short-term inulin intake stimulates the absorption of both Ca and Mg. Furthermore, not only these results confirmed that Ca absorption declines considerably with age but also showed for the first time that Mg absorption also declines with age in the rat. Moreover, these results confirmed our hypothesis of a greater stimulatory effect of inulin on Ca absorption in aged rats than in the young or adult rats, and a similar stimulatory effect of inulin on Mg absorption in aged rats and young and adult rats. Further studies are required to explore this effect on longer inulin intake periods and to validate these results on the stimulatory effect of inulin on Ca and Mg absorption in the elderly.

## Abbreviations

Ca: calcium; Mg: Magnesium; ICP/MS: Inductively coupled plasma/mass spectrometry, OS: oligosaccharides; SCFA: Short-chain fatty acids;
